# Supplementary material for: Change in nutritional status of urban slum children before and after the first COVID-19 wave in Bangladesh: A repeated cross-sectional assessment
Source: PLOS Glob Public Health. 2022 Jul 1;2(7):e0000456. doi: 10.1371/journal.pgph.0000456 (PMC10021417; doi:10.1371/journal.pgph.0000456)
Supplement: S1 Table — (DOCX) [file pgph.0000456.s002.docx]

**Supporting information 2**

**S2 Table:** Sampling distributions at baseline and endline

|  | **Baseline (2020)**  *N*=346 | | **Endline (2021)**  *N*=773 | |
| --- | --- | --- | --- | --- |
| **Korail Slum (Dhaka)** | | | | |
| No. | Cluster Code | Cluster Size  (*n* sampled/ cluster) | Cluster Code | Cluster Size  (*n* sampled/ cluster) |
| 1 | K1 | 4 | K1 | 42 |
| 2 | K2 | 1 | K2 | 42 |
| 3 | K3 | 2 | K3 | 37 |
| 4 | K4 | 25 | K4 | 18 |
| 5 | K5 | 24 | ~~-~~ | - |
| 6 | K6 | 20 | K6 | 38 |
| 7 | K8 | 17 | K8 | 42 |
| 8 | K9 | 43 | K9 | 42 |
| 9 | K11 | 31 | K11 | 42 |
| 10 | - | - | K12 | 42 |
| Total | 9 | *n*=167 | 9 | *n*=345 |
| **Tongi Slum (Gazipur)** | | | | |
| No. | Cluster Code | Cluster Size  (*n* sampled/ cluster) | Cluster Code | Cluster Size  (*n* sampled/ cluster) |
| 1 | T1 | 31 | T1 | 42 |
| 2 | T2 | 29 | T2 | 37 |
| 3 | T3 | 30 | T3 | 42 |
| 4 | T4 | 24 | T4 | 42 |
| 5 | T5 | 29 | T5 | 42 |
| 6 | T6 | 16 | T6 | 42 |
| 7 | T7 | 20 | T7 | 42 |
| 8 | - | - | T8 | 42 |
| 9 | - | *-* | T9 | 42 |
| 10 | - | *-* | T10 | 29 |
| 11 |  | *-* | T11 | 26 |
| Total | 7 | *n*=179 | 11 | *n*=428 |
