# Supplementary figures and images for: Change in nutritional status of urban slum children before and after the first COVID-19 wave in Bangladesh: A repeated cross-sectional assessment
Source: PLOS Glob Public Health. 2022 Jul 1;2(7):e0000456. doi: 10.1371/journal.pgph.0000456 (PMC10021417; doi:10.1371/journal.pgph.0000456)

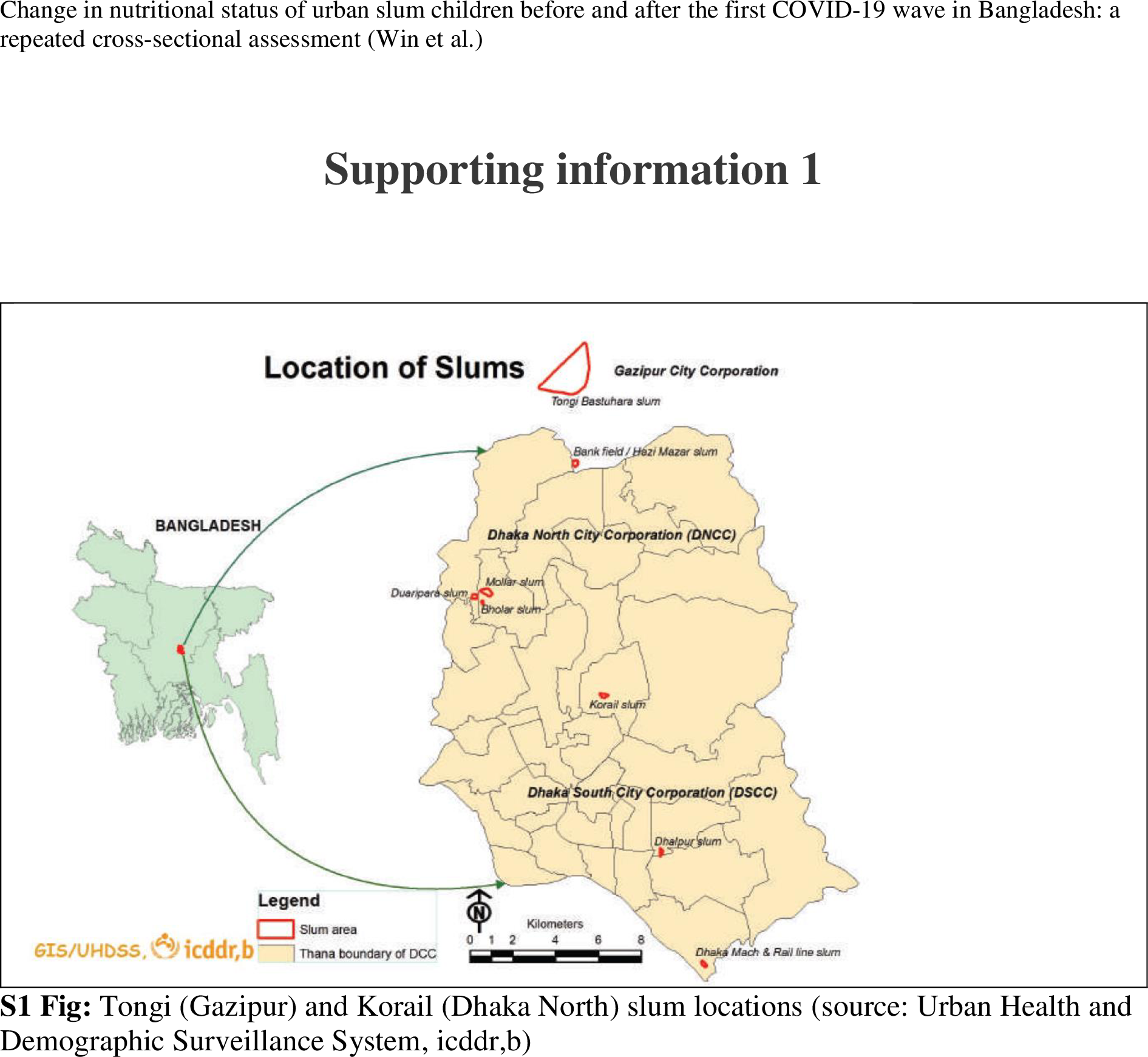

Supplement: S1 Fig — (TIF) [file pgph.0000456.s003.tif]

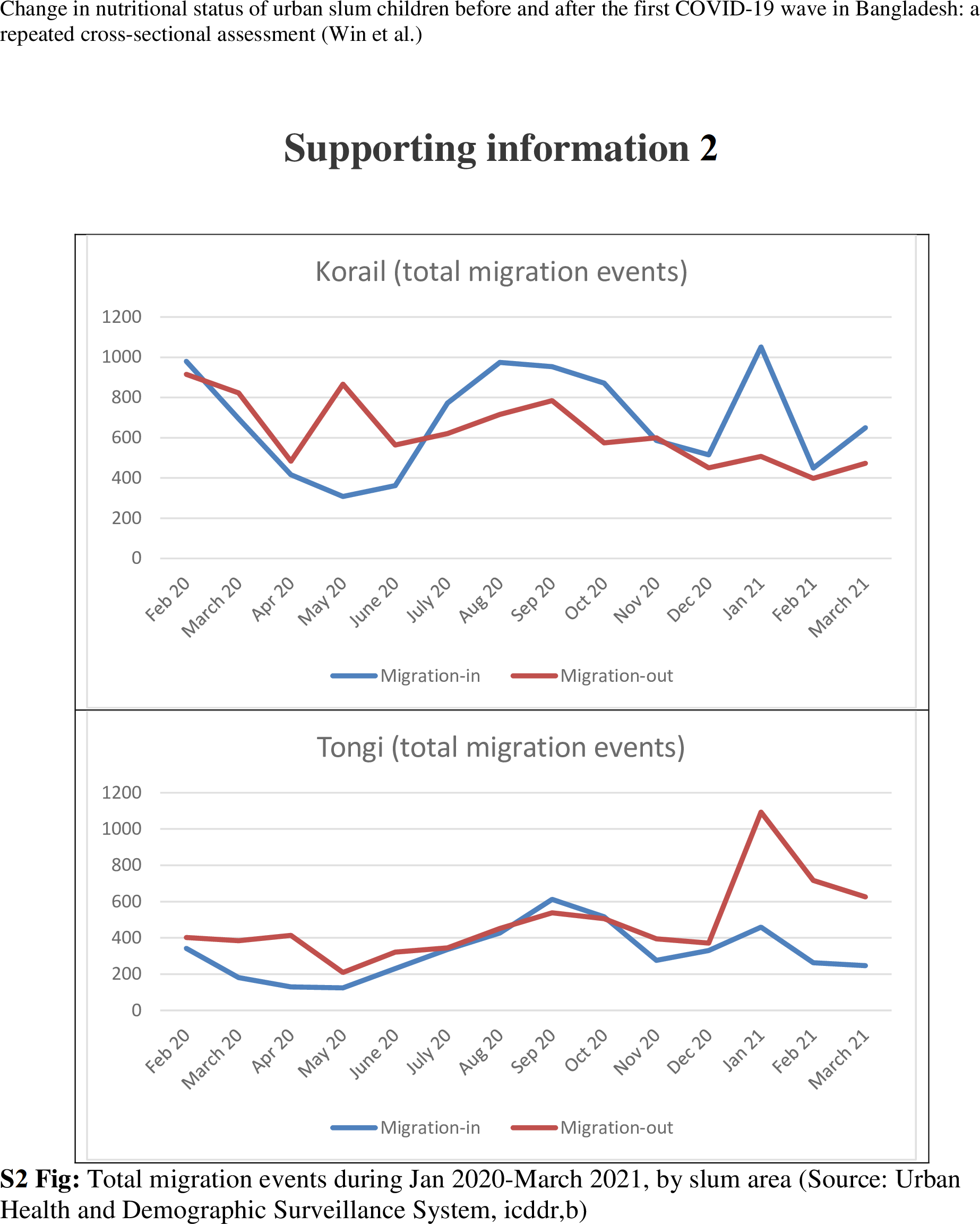

Supplement: S2 Fig — (TIF) [file pgph.0000456.s004.tif]

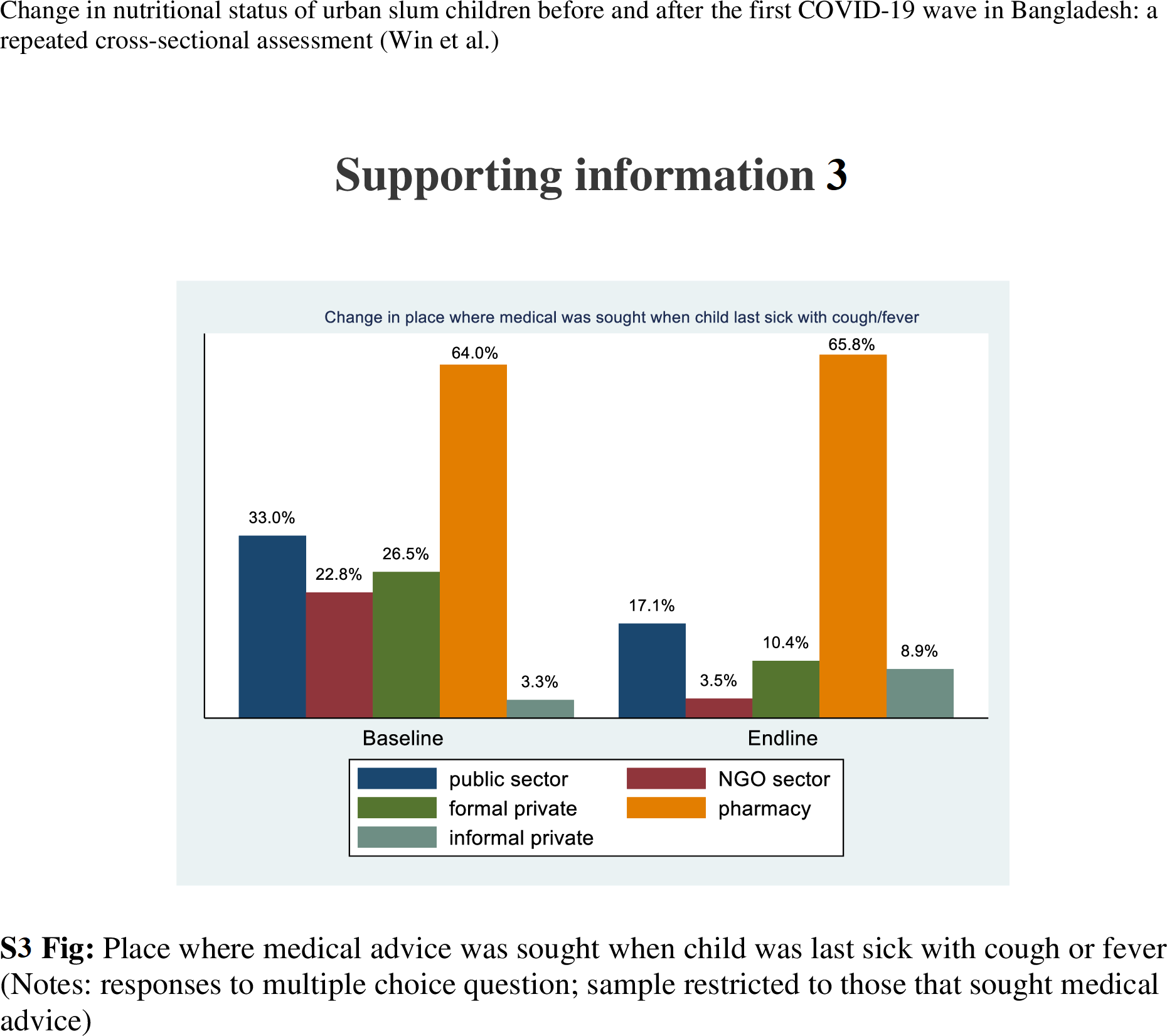

Supplement: S3 Fig — (TIF) [file pgph.0000456.s005.tif]
