# Supplementary material for: Change in nutritional status of urban slum children before and after the first COVID-19 wave in Bangladesh: A repeated cross-sectional assessment
Source: PLOS Glob Public Health. 2022 Jul 1;2(7):e0000456. doi: 10.1371/journal.pgph.0000456 (PMC10021417; doi:10.1371/journal.pgph.0000456)
Supplement: S4 Fig — (DOCX) [file pgph.0000456.s006.docx]

**Supporting information 4**

| (A) | (B) |
| --- | --- |
| (C) | (D) |
| (E)  | (F) |
| (G)(I) | (H)(J) |

**S4 Fig:** Kernel density estimates of HAZ and WHZ distributions (panels A and B: full sample; panels C-J: by subgroup)
