# Supplementary material for: Change in nutritional status of urban slum children before and after the first COVID-19 wave in Bangladesh: A repeated cross-sectional assessment
Source: PLOS Glob Public Health. 2022 Jul 1;2(7):e0000456. doi: 10.1371/journal.pgph.0000456 (PMC10021417; doi:10.1371/journal.pgph.0000456)
Supplement: Attachment — Submitted filename: Response to Reviewers.docx [file pgph.0000456.s007.docx]

Response to Reviewers

Please see below point-by-point responses to review comments with changes highlighted in the revised manuscript:

**Journal requirements:**

1. Please include a complete copy of PLOS’ questionnaire on inclusivity in global research in your revised manuscript. Our policy for research in this area aims to improve transparency in the reporting of research performed outside of researchers’ own country or community. The policy applies to researchers who have travelled to a different country to conduct research, research with Indigenous populations or their lands, and research on cultural artefacts. The questionnaire can also be requested at the journal’s discretion for any other submissions, even if these conditions are not met.

**Response:** A completed version of the questionnaire on inclusivity in global research has been included with the revised manuscript and uploaded as Supporting Information (S2 Checklist). The reference is also included on p.7 (line 134-136) of the revised manuscript.

2. In the online submission form, you indicated that your data is available only on request from a third party. Please note that your Data Availability Statement is currently missing [the name of the third party contact or institution / contact details for the third party, such as an email address or a link to where data requests can be made]. Please update your statement with the missing information.

**Response:** The data availability statement has been updated with contact information.

3. Please amend your detailed Financial Disclosure statement. This is published with the article, therefore should be completed in full sentences and contain the exact wording you wish to be published.

**Response:** Financial Disclosure statement has been updated.

**Editor’s Comments**

4. The present article has important information to understand the impact of COVID-19 on child nutritional status. However, I have a major concern regarding the ethical issues related to the first survey undergone before lockdown. Hence, a detail description of COVID protocol has to be provided. Information how subjects are protected from COVID during the survey has to be included.

**Response:** Thank you for raising this concern and allowing us to clarify this in the revised manuscript. The first survey started well before any restrictions and reporting of the first COVID-19 case in Bangladesh; and the fieldwork was immediately suspended when the government announced plans for “nationwide holidays” as precautionary means to control further spread of the disease during early stages of the pandemic. Resumption of field activities after one year followed after careful consideration of local epidemiological context (when daily case positivity rates were dropping) and passing various institutional review processes/ clearances (within the local research institution (icddr,b), government counterpart (UPHCSDP), and ADB country office). Implementation of field activities followed strict COVID-19 safety protocols to protect the field staff and the community at large, and the second survey was suspended with the onset of a second wave. We have provided the clarifications, along with details of COVID-19 safety protocols in place during the survey, on pp. 6-7 of the revised manuscript.

**Reviewers’ comments**

**Reviewer #1:**

5. The MS is actual and new. It can be recommended for publication. The results are corresponded to COVID-19 pandemic. The article demonstrated how nutrition status of children is changing due to pandemic.

**Response:** Thank you for the kind review.

**Reviewer #2:**

6. Thank you for conducting the cross-sectional study related to the nutritional status of children in Bangladesh during the pandemic. The study results are useful for the government, healthcare providers, policymakers, etc. for formulating different strategies in their fields. I have the following suggestions for the manuscript. The comments are listed below.

**Response:** Thank you for the kind review and the additional suggestions – we really appreciate them and have done our best to address them all as further outlined below.

*Data collection and ethical approval:*

7. Provide the calculation and determination of the sample size.

**Response:** The target sample size calculation is now included on page 5 (lines 98-101) of the revised manuscript.

8. Specify the questionnaires are self-designed or adopted from another study. Provide detailed information related to the questionnaire used in the current study. For example, if the questionnaires are self-designed, state the information related to the validity and reliability of the questionnaires, and any professional to validate the questions. If it is adopted from other studies, please state the questionnaires are free to use or approval have been obtained.

**Response:** The questions were adapted from publically available sources and pre-tested in the field; we have added this information in the revised manuscript on p. 6 (lines 119-122).

*Limitations*

3. Provide a sentence explaining the solution related to each limitation stated in this section.

**Response:** Thank you for the suggestion. We have added respective potential solutions in the limitation section on pp.19-20 of the revised manuscript.

*Others*

4. Suggested to provide a research checklist (e.g. STROBE statement for reporting a cross-sectional study) as supplementary information (OPTIONAL).

**Response:** Thank you for the suggestion. We were guided by the STROBE checklist in preparation of the manuscript but opted not to include it as a separate file.
